# Population (Antibody) Testing for COVID-19—Technical Challenges, Application and Relevance, an English Perspective

**DOI:** 10.3390/vaccines9060550

**Published:** 2021-05-24

**Authors:** Peter A. C. Maple

**Affiliations:** 1 Clinical Neurology Research Group, Department of Neurology, Division of Clinical Neuroscience, University of Nottingham School of Medicine, Queen’s Medical Centre, Nottingham NG7 2UH, UK; peter.maple@nottingham.ac.uk; 2 Molecular (COVID) Department, UK Lighthouse Laboratory, Cheshire SK10 4TG, UK

**Keywords:** COVID-19, SARS-CoV-2, antibodies, immunity, seroepidemiology, England

## Abstract

In the UK, population virus or antibody testing using virus swabs, serum samples, blood spots or oral fluids has been performed to a limited extent for several diseases including measles, mumps, rubella and hepatitis and HIV. The collection of population-based infection and immunity data is key to the monitoring of disease prevalence and assessing the effectiveness of interventions such as behavioural modifications and vaccination. In particular, the biological properties of severe acute respiratory syndrome coronavirus-2 (SARS-CoV-2) and its interaction with the human host have presented several challenges towards the development of population-based immunity testing. Measuring SARS-CoV-2 immunity requires the development of antibody assays of acceptable sensitivity and specificity which are capable of accurately detecting seroprevalence and differentiating protection from non-protective responses. Now that anti-COVID-19 vaccines are becoming available there is a pressing need to measure vaccine efficacy and the development of herd immunity. The unprecedented impact of the SARS-CoV-2 pandemic in the UK in terms of morbidity, mortality, and economic and social disruption has mobilized a national scientific effort to learn more about this virus. In this article, the challenges of testing for SARS-CoV-2 infection, particularly in relation to population-based immunity testing, will be considered and examples given of relevant national level studies.

## 1. Introduction

Towards the end of 2019, Chinese authorities identified several cases of viral pneumonia of unknown cause apparently linked to a seafood market in Huanan, Hubei Province [1]. The agent responsible was subsequently shown to be a novel coronavirus and was named novel coronavirus 2019, nCoV [2]. Further taxonomic studies [3] designated the novel coronavirus as severe acute respiratory syndrome-2 coronavirus (SARS-CoV-2), subgenus *Sarbecovirus*, genus *Betacoronavirus*. The clinical syndrome associated with infection by SARS-CoV-2 was officially termed COVID-19 and on 11 March 2020 a pandemic of COVID-19 was declared by the World Health Organization [4]. In the UK, in response to the emergence of SARS-CoV-2, the International Severe Acute Respiratory and Emerging Infections Consortium (ISARIC) World Health Organization (WHO) Clinical Characterization Protocol UK (CCP-UK) study was initiated 17 January 2020 [5]. The first confirmed case of COVID-19 in the UK was reported on 31 January 2020 [6] and within a few months, hospitals in England, Scotland and Wales had admitted 59,215 patients with COVID-19 [5]. The UK COVID-19 epidemic had begun and despite the best efforts of the UK Governments (England, Scotland, Wales and Northern Ireland) over the 12 months since the first reported case there have been approximately 3.8 million notified cases and 125,000 deaths [7]. In this paper, the challenges of antibody testing for SARS-CoV-2 infection will be considered followed by an overview of the results of several English studies to chart the COVID-19 epidemic. Finally, the contributions and potential limitations of population antibody testing for SARS-CoV-2 infection surveillance and monitoring the impact of control measures will be assessed. 

## 2. Estimating the Incidence of SARS-CoV-2 Infection

**Figure 1:**
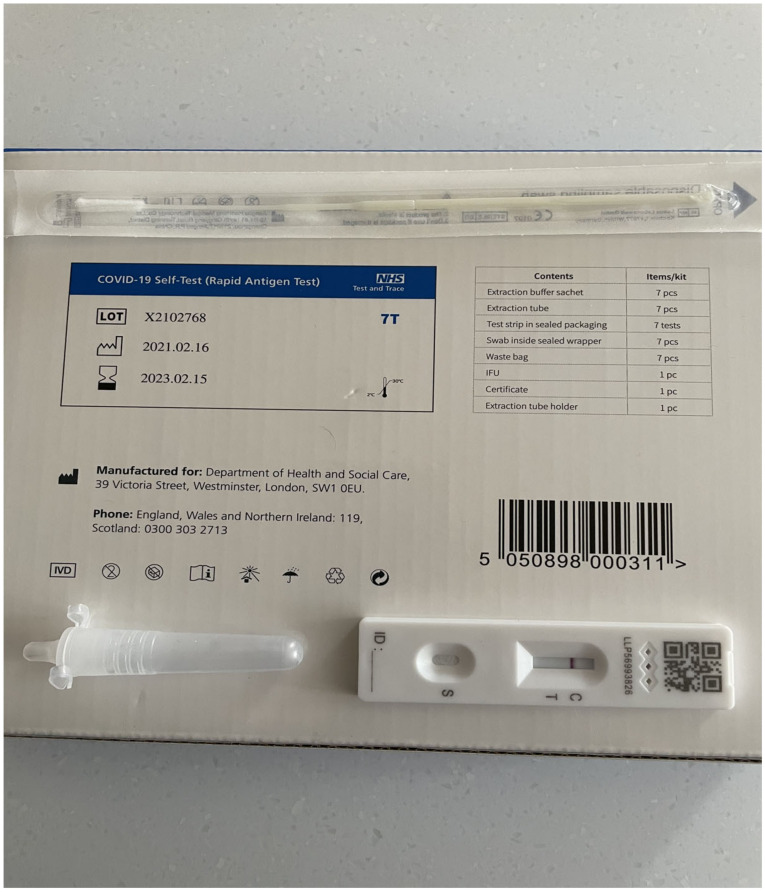
A SARS-CoV-2 antigen home testing kit.

The mainstay or recognized Gold Standard of COVID-19 confirmation is the detection of SARS-CoV-2 nucleic acid in appropriately taken specimens [8,9]. Typically, polymerase chain reaction (PCR) methods have been used; however, other methodologies such as loop mediated isothermal amplification (LAMP) have given promising results [9,10]. Virus antigen tests using lateral flow technology are also widely available offering the means to screen for infection in non-laboratory settings in real time [11,12]. Virus antigen lateral flow devices (Figure 1) can be mass produced at low cost, but their sensitivity and specificity are generally lower than those of nucleic acid detection methodologies [13,14]. The detection of viral RNA using real-time PCR has variable sensitivity depending on the type of clinical specimens collected, the protocol used [9,15] and the time of sampling. For instance, Chen and colleagues [15] have reported the average duration of SARS-CoV-2 viral shedding to be 17.3 days in hospitalized patients with 12.1 days for ward patients compared to 24.4 days patients in intensive care. The viral load detected is also dependent upon the clinical condition of the patient with lower viral loads detected in asymptomatic patients compared to patients with pulmonary signs and symptoms [16,17]. Long-term virus shedding has also been documented in asymptomatic or mildly symptomatic patients as well as those with severe disease [15,16,17].

## 3. Evolution of the COVID-19 Epidemic and Testing Strategies in England

**Figure 2:**
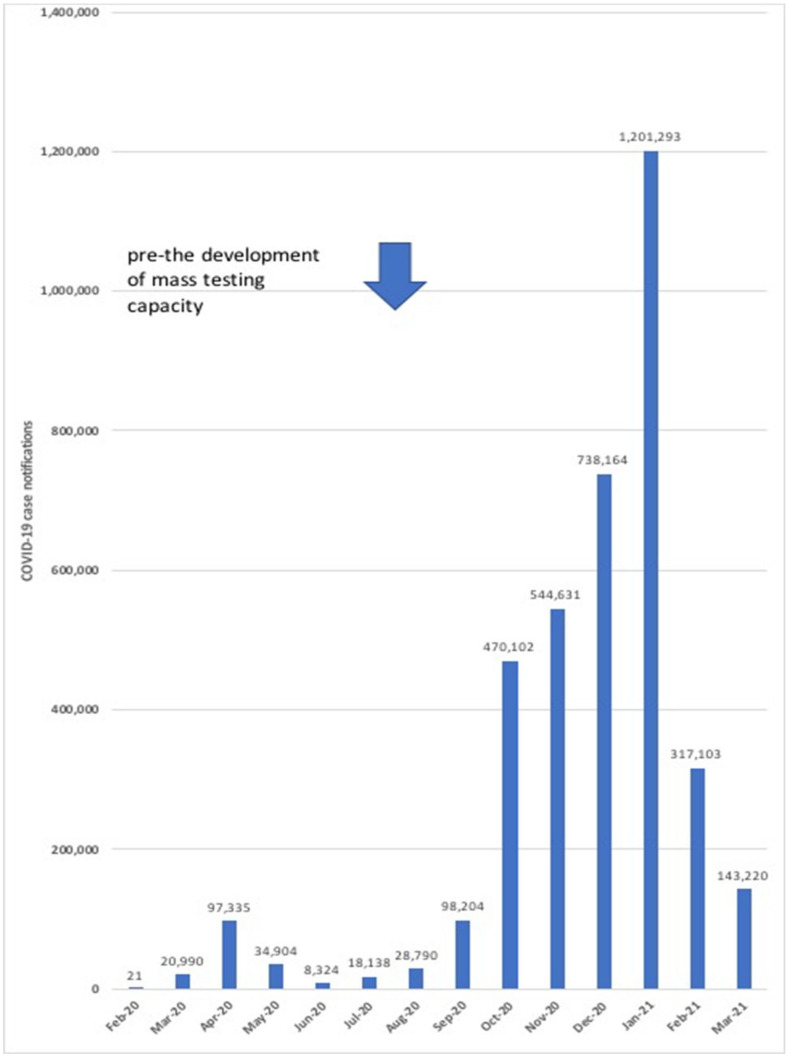
Monthly COVID-19 case notifications for England.

The evolution of the COVID-19 epidemic in England is shown in Figure 2 and over the 12 months since the first reported case, there have been approximately 3.8 million notified cases and 125,000 deaths [7]. When assessing these data, factors such as the availability of testing and the extent of asymptomatic infection are potentially significant confounders. The early weeks of the English COVID-19 epidemic were characterized by an urgent need to establish increased testing capacity [18] which was initially targeted at hospital patients with severe respiratory illness. On 4 April 2020 the English Government’s Department of Health and Social Care published a document [19] titled “*Coronavirus (COVID-19) scaling up our testing programmes*” which outlined the components of a national testing strategy (Table 1). In an attempt to radically increase test capacity expressions of interest were invited from the private sector and several strategic alliances were formed, for example, the UK lighthouse laboratory network.

## 4. Virological Properties of SARS-CoV-2

There are seven human coronaviruses, two (HCoV-229E and HCoV-NL63) belong to the genus Alphacoronavirus and the remainder are classified within the genus Betacoronavirus [3]. There are several subgenera within the genus Betacoronavirus including the Embecoviruses (HCoV-OC43 and HCoV-HKU1), the Sarbecoviruses (SARS-CoV and SARS-CoV-2) and the Merbecovirus subgenus (MERS-CoV). The human alphacoronaviruses and the human Embecoviruses have been linked with outbreaks of lower and upper respiratory tract infections and are considered to be common cold viruses [20,21].

Human coronaviruses are enveloped, single-stranded RNA viruses, 120–160 nm in diameter, and on electron microscopy [22] a characteristic crown (corona) is seen due to a fringe of petal-shaped surface projections (peplomers/spikes). They have a relatively large non-segmented genome of approximately 30 kb length which encodes four main structural proteins (the spike surface glycoprotein—S, the small envelope protein—E, the matrix protein—M and the nucleocapsid protein—N) and in the case of SARS-CoV-2, 15 non-structural proteins and eight accessory proteins [23]. The spike protein is a trimeric class 1 fusion protein, comprising two subunits, which form stalk (S2) and petal projections (S1) on the surface of the virion [24]. The S1 subunit contains a receptor binding domain which in the case of SARS-CoV-2 [25] is responsible for high affinity binding to the host cell receptor angiotensin converting enzyme 2 (ACE2). The S2 subunit functions in membrane fusion and virus entry by establishing fusion peptides and in SARS-CoV-2 a novel furin highly conserved cleavage site has been identified at the S1/S2 interface [26]. The spike protein has been shown to be a key determinant for virus immunogenicity, pathogenicity and transmissibility [27]. The nucleocapsid protein has a primary role of packaging the viral genome; however, it is a multifunctional protein having involvement in several processes including virus assembly, virus budding, and modulation of the host immune response [28,29].

There is significant evidence [30,31] that the Sarbecoviruses are evolutionary descended from bat coronaviruses. It has been proposed that transmission to humans has been achieved via intermediary hosts—the palm civet for SARS-CoV and the Malayan pangolin for SARS-CoV-2. Phylogenetic studies [32] have reported that the SARS-CoV-2 genome shares >90% nucleotide identity with the genomes of bat coronaviruses RaTG13 and RmYN02, and 80% identity with the genome of SARS-CoV. Although this is compelling evidence in support of the aforementioned evolutionary hypotheses, there has been some discussion [33] that other processes may have been involved in the generation of SARS-CoV-2. In comparison with other RNA viruses, SARS-CoV-2 has a limited capacity to mutate [34]; however, several new variants of concern have evolved [35]. In particular, spike protein mutations can lead to an increase in virus transmissibility and potentially increased virulence prompting the need for enhanced control measures [36].

### 4.1. SARS-CoV-2 Infection and the Immune Response

The clinical manifestations of SARS-CoV-2 infection range from asymptomatic/mild [37] to viral pneumonia and severe acute respiratory syndrome [38] together with the development of complications including cardiovascular events (e.g., heart failure, thromboembolism), neurological manifestations, and hyperimmune immune activity/cytokine storm [39]. Infection by SARS-CoV-2 elicits both innate and adaptive immune responses which may differ depending upon the clinical course of disease—a weak and transient immune response in asymptomatic infection [40] versus a hyperinflammatory (hypercytokinaemic) immune response in severe infection [41]. The innate immune response plays a key role in fighting COVID-19 infection yet contributes also to disease progression [42]. In the context of this article, measuring the innate immune response does not lend itself to COVID-19 diagnosis or surveillance at a population level due to its lack of specificity and the complexity of the laboratory methods involved and will not be discussed further. The COVID-19 adaptive immune responses, comprising cellular and humoral components have vital roles in limiting virus proliferation and spread which have been comprehensively reviewed elsewhere [43,44]. In brief, IgG and IgM are detectable from one to three weeks following the onset of symptoms [45]; however, many factors (e.g., antigen and assay method, severity of infection, presence of pre-existing immunity) contribute to the variability of time of seroconversion and its detection during this time period. Similarly, specific T-cell responses to nucleocapsid and spike proteins develop within a few weeks following the onset of symptoms and these have been shown to correlate with the magnitude of the humoral response and to be associated with protection over the longer term [46,47]. The immune correlates of protection require careful interpretation as Sui and colleagues have suggested [48]. Essentially, higher viral loads and more robust and longer lasting immune responses are seen in cases of severe COVID-19 and lower viral loads and weaker immune responses which decline more quickly are seen in cases of mild or asymptomatic COVID-19.

### 4.2. Diagnostic and Seroprevalence Testing for SARS-CoV-2 Infection

Measuring the adaptive immune response underpins the diagnosis and surveillance of many infectious diseases; however, there are several aspects of SARS-CoV-2 biology to be considered when developing assays for COVID-19. The spike protein and nucleocapsid of SARS-CoV-2 are both immunogenic and there are pros and cons for the use of either antigen in antibody detection assays. The nucleocapsid protein is produced in large amounts, is highly conserved, and high levels of specific antibody have been reported [49] in COVID-19 patients. The spike protein is significantly larger than the nucleoprotein and the recombinant antigens used in laboratory assays are usually based around the receptor binding domain. Antibodies binding to the receptor binding domain have neutralizing potential [50] and are considered a better marker for protection than nucleocapsid antibodies. 

In response to the SARS-CoV-2 pandemic, there has been an urgent need to develop and manufacture new virus commercial antibody assays and regulatory bodies have revised the usual approval mechanisms for new commercial kits; for example, by the introduction of emergency use authorization [51]. Public Health England have performed several evaluations of SARS-CoV-2 antibody assays [52] in an attempt to standardize the data relating to the performance characteristics of such assays (Table 2). Unfortunately, direct comparisons of assay performance cannot be made due to differences in the constitution of the sample panels used in each evaluation. Furthermore, important considerations in the evaluation of SARS-CoV-2 antibody assays are the potential for spectrum bias [53] and the general lack of availability of a Gold Standard comparator assay (neutralization assay) due to its technical complexity [54]. This has resulted in a clinical history and SARS-CoV-2 polymerase chain reaction positivity been used to categorize sera as SARS-CoV-2 infection true positives in many evaluations.

SARS-CoV-2 antibody assays are available in two fundamentally different formats; either as traditional laboratory-based immunoassays (e.g., ELISA) or immunochromatographic based lateral flow assays [55]. The latter can be readily manufactured as point of care testing devices which can be used by untrained individuals and because results are usually available within thirty minutes of adding the sample they are also known as rapid test devices. Such devices may not be suitable for assessing individual protection [56], but they have been considered for use in serosurveillance studies in England [57] despite reports [58,59] of variable performance.

SARS-CoV-2 nucleic acid testing by methods such as polymerase chain reaction is the recommended methodology for confirmation of a COVID-19 diagnosis because specific antibody production is usually not detectable until two to three weeks following the onset of symptoms [44,60]. Serological detection of SARS-CoV-2 infection [61,62] can be used to retrospectively confirm a diagnosis of COVID-19 in previously symptomatic individuals who were infected several weeks/months previously and were either not tested by PCR or had become PCR negative. Furthermore, positive SARS CoV-2 serology can confirm infection in individuals who were asymptomatic or mildly symptomatic who have never presented for PCR testing (or tested negative). Consequently, estimates of the prevalence of SARS-CoV-2 infection can be significantly higher when based upon serological testing compared to case presentation and/or virus nucleic acid detection data. As the production of IgM is short-lived [44,61], detection of specific IgG is the marker of choice for SARS-CoV-2 seroprevalence surveys. When undertaking such surveys, it is important to give due consideration to the variable decay of SARS-CoV-2 IgG and the fact that detection of specific antibody does not necessarily correlate with protective immunity as previously mentioned. Several studies [40,62,63] have reported significant losses of specific IgG/neutralizing antibodies three to six months following SARS-CoV-2 infection. In contrast, other studies [64,65,66] have shown SARS-CoV-2 sero-positivity to be mostly stable up to six months.

## 5. Population Antibody Screening for SARS-CoV-2 Infection Undertaken by the UK Government in England

The UK Government has sponsored several population-based SARS-CoV-2 antibody surveillance studies within England [67] which are summarized in Table 3. One of the largest studies has been the COVID-19 infection survey which uses repeat household visits to establish the proportion of the general population who have COVID-19 [68]. The number of participants has increased over time and a target has been set of testing 150,000 every two weeks from October 2021. Volunteers have swabs taken for SARS-CoV-2 nucleic acid testing and a proportion also supply blood (venous or finger-prick) for antibody testing. SARS CoV-2 antibodies to spike and nucleoproteins are detected using validated commercial and in-house immunoassays [69]. In-house assays have been used for spike protein antibodies including time-resolved fluorescence immunoassay and enzyme-linked immunosorbent assay (ELISA). According to this survey as of March 2021, it is estimated one in three people in England have either experienced SARS-CoV-2 infection or have been vaccinated [70]. Another survey, which has run since May 2020, is the Real-time assessment of community transmission (REACT-2) study. In this study [71], home-based antibody testing using lateral flow devices [58] has been used to assess SARS-CoV-2 antibody prevalence over several time periods for people residing in England. A recent cross-sectional community survey [72] undertaken between 26 January 2021 and 8th February 2021 involving 172,099 people has estimated SARS-CoV-2 antibody seroprevalence to be 13.9% (95% CI: 13.7–14.1). Other studies have investigated SARS-CoV-2 antibody dynamics in selected populations. For instance, the Sarscov2 Immunity and Reinfection Evaluation (SIREN) study has investigated whether prior infection with SARS-CoV-2 protects against reinfection. In this prospective longitudinal cohort study, healthcare workers have been sampled every two to four weeks over a 12-month period [73]. Over the period 18 June 2020 to 31 December 2020, a total of 30,625 participants had been enrolled and an 84% protective effect resulting from past infection reported [74]. In a separate study (VIVALDI), the extent of SARS-CoV-2 infection has been investigated for long term care facilities and follow-up testing of care home residents and staff has been performed to estimate the protective effect of SARS-Cov-2 infection [75]. In a sample of 682 residents and 1429 staff from 100 care homes, it was determined that the presence of IgG antibodies to nucleocapsid was associated with a substantially reduced risk of reinfection [76]. Several other studies are also underway, such as infection surveys of schools [77,78] and cellular immunity surveys of healthcare workers following natural infection and vaccination [79,80].

## 6. The Challenges of SARS-CoV-2 Population Antibody Testing

England, primarily through the activities of the Public Health Laboratory Service which following reorganization was incorporated into the Health Protection Agency which subsequently became Public Health England has a tradition of conducting population antibody studies for communicable diseases [81]. Examples include surveys of diphtheria, tetanus and pertussis [82,83], measles, mumps and rubella [84], and hepatitis/HIV virus infection prevalence [85,86]. Typically, these sero-surveys were underpinned by the availability of suitable validated reference assays and reference sera, the assays used had high sensitivity and specificity, and the antibodies detected showed long term stability. In contrast, with SARS-CoV-2 validated reference assays (e.g., neutralization assay) and reference sera have not generally been available, and variable assay sensitivity and specificity has been reported. Furthermore, specific SARS-CoV-2 antibody levels may decay significantly over short periods of time and the extent of decay can vary with the assay used [87]. Protective antibody levels remain to be established. Added to this list of challenges is the capacity of SARS-CoV-2 to mutate and for new variants to quickly increase circulation in populations [88,89]. Some of these variants [90,91] may have different immune reactivities compared to other circulating or displaced strains. Finally, the lack of established reference assays and reference sera means that there is no common denominator for antibody responses in the increasing number of reports of the efficacy of new SARS-CoV-2 vaccines. 

Table 3 lists examples of population-based studies performed in England. Many of these studies are ongoing and evolving to answer new questions as national circumstances and priorities and control measures change. The table lists examples as it is not possible to provide a definitive list as new projects are frequently starting, and a comprehensive central database is not available in the public domain as far as the author is aware. The website links provided [67,68,70,77,78,79,80] are a valuable source of current information. Examples of projects not included in Table 3 include a raft of studies relating to paediatric surveillance and COVID-19 to answer questions including the risk of vertical transmission during pregnancy (periCOVID) and assessing the long-term impact of COVID-19 in children and young people with Long COVID (CLoCK) [92,93,94,95]. As new SARS-CoV-2 vaccines [96,97] become available and new variants emerge there will be a continuing need to assess the extent and degree of protective immunity within populations. 

## 7. Final Comments

Therefore, much has happened in the last year, not only in England, but worldwide. Not only has the COVID-19 pandemic severely impacted upon population health in terms of morbidity and mortality, it has also widely disrupted economic and healthcare systems. In England, there has been a need to introduce measures limiting individual freedoms or movement including the imposition of national lockdowns and requirements to “*stay at home*”, wear face masks in designated areas and limit social gatherings. The first English national lockdown commenced late March 2020 and measures were relaxed from mid-May [98]. Figure 2 demonstrates the impact of these policies with COVID-19 cases troughing by June 2020; unfortunately, it also shows the outcome of this relaxation with subsequent national lockdowns implemented November 2020 (four weeks) and January 2021. The economic and social turmoil following the emergence of SARS-CoV-2 has been unprecedented. Matched against this has been an unparalleled scientific effort to learn more about SARS-CoV-2. At the time of writing, following the development of COVID-19 vaccines and the beginning of mass population vaccination we are now moving from a pre-vaccination era to a vaccination era. The free exchange of scientific information is absolutely vital to our efforts to limit the impact of COVID-19. Much of the work undertaken by the UK Government has been documented; for example, on the Internet and communicated via the scientific press. This article is based upon these forms of communication and the efforts of many contributors ranging from clinical trial participants, funding bodies, healthcare and scientific workers, all of whom have contributed to advances in our knowledge and practise in relation to COVID-19. COVID-19 is a Worldwide problem and requires a Worldwide response. 

## Figures and Tables

**Table 1:** Components of the English national testing strategy for COVID-19 as described April 2020 [19].

Testing Programme	Target	Delivery Mechanism
Pillar one	Scaling up National Health Service virus swab testing for those with a medical need and, where possible, the most critical key workers.	Testing to be performed by National Health Service and Public Health England laboratories.
Pillar two	Mass-virus swab testing for critical key workers in the National Health Service, social care and other sectors.	Commercial/private partnerships initiated to deliver a mass testing capability.
Pillar three	Mass antibody testing to help determine if people have immunity to coronavirus.	Once satisfactory commercial tests identified proposed home testing using finger prick samples.
Pillar four	Surveillance testing to learn more about the disease and help develop new tests and treatments.	National surveillance programmes for population blood testing, using high accuracy antibody testing delivered by designated laboratories.
Pillar five	Spearheading a diagnostics national effort to build a mass-testing capacity at a completely new scale.	Working with commercial/industrial partners to deliver high volume testing capacity as part of a resilient, diagnostic capability.

**Table 2:** Summary of SARS-CoV-2 commercial antibody assay kit performance studies conducted by Public Health England, UK.

Commercial Assay	Samples Tested *	Sensitivity (95% CI)	Specificity (95% CI)
Beckman Coulter Access Anti-SARS-CoV-2 IgG assay	100 pos, 499 neg	76.5% (66.0–85.0)	99.3% (97.8–99.8)
Siemens Atellica-IM SARS-CoV-2 Total (COV2T) assay	100 pos, 499 neg	89.4% (80.8–95.0)	100% (99.1–100)
Ortho Clinical Diagnostics Vitros Anti-SARS -CoV-2 assay	100 pos, 491 neg	91.8% (83.8–96.6)	99.5% (98.2–99.9)
DiaSorin LIAISON SARS-CoV-2 S1/S2 IgG assay	100 pos, 472 neg	69.4% (58.5–79.0)	97.7% (95.8–99.0)
Euroimmun Anti-SARS-CoV-2 ELISA (IgG) assay	93 pos, 499 neg	73.4% (62.3–82.7)	99.0% (97.5–99.7)
Abbott SARS-CoV-2 IgG assay	96 pos, 760 neg	93.9% (86.3–98.0)	100% (99.1–100)
Roche Elecsys Anti-SARS-CoV-2 EIA	93 pos, 472 neg	86.1% (76.5–92.8)	100% (99.1–100)
Siemens Atellica-IM SARS-CoV-2 (sCOVG) assay	115 pos, 500 neg	72.5% (61.4–81.9)	100% (98.9–100)

* Convalescent SARS-CoV-2 blood samples collected 14 days or longer following symptom onset. Data are unique for each assay as the samples used were not all the same. The data used are as published by Public Health England and are available on the website http://www.gov.uk/government/publications/COVID-19-laboratory-evaluations-of-serological-assays#history (accessed on 10 March 2021) [52].

**Table 3:** Examples of SARS-CoV-2 antibody population surveys undertaken in England [67].

Study Protocol	Assay Used/Samples Tested	Observations
Office for National Statistics COVID-19 Infection Survey. Commenced April 2020 [68].	Commercial and in-house assays used [69]. Target of testing 150,000 people in two-week periods from October 2020.	In England, as of March 2021, it is estimated that 34.6% (95%CI: 34.0–35.3) of individuals have antibodies against SARS-CoV-2 [70].
Imperial College. Real-time assessment of community transmission (REACT) study—2. Commenced May 2020 [71].	Lateral flow assays for home—based testing [58]. Approximately 150,000 volunteers tested every six weeks.	In round 5 [72] conducted between 26 January 2021 and 8 February 2021, sera were tested from 155,172 people and the overall SARS-CoV-2 seroprevalence was 13.9%.
Public Health England. Sarscov2 immunity and Reinfection Evaluation (SIREN) study [73].	Commercial and in-house assays used. Target of recruiting 100,000 healthcare workers with samples collected over a 12-month period.	A prior history of SARS-CoV-2 infection was associated with an 84% lower risk of reinfection [74].
COVID-19 surveillance study in care homes (Vivaldi 1 and Vivaldi 2)—University College London and University of Birmingham and others [75].	Abbott ARCHITECT SARS-CoV-2 IgG (nucleoprotein) and MSD V-Plex COVID-19 assay (76). Staff and residents of 105 care homes. Target 5000 residents and 6500 staff.	In a sample of 682 residents and 1429 staff, the presence of IgG antibodies to nucleocapsid was associated with a substantially reduced risk of reinfection for up to 10 months following primary infection [76].
Schools Infection Survey -the London School of Hygiene and Tropical Medicine, Public Health England, and the Office for National Statistics	Finger prick and oral fluid samples tested for antibodies using commercial (Roche cobas Elecsys anti-SARS-CoV-2 assay) and in-house assays [77].	In the second round of testing, 7089 pupils and 5114 staff in 121 schools were tested. Approximately, 15% of staff were SARS-CoV-2 antibody positive [78].
Protective immunity from T-cells in healthcare workers (PITCH)—Several UK universities [79].	Peripheral blood mononuclear cells tested by IFNgamma ELIspot assay. Target of recruiting 2000 healthcare workers.	In a study of 237 healthcare workers following a single dose of the Pfizer BN2162b2 vaccine T cell responses were six-fold higher in vaccine recipients at 28 days post vaccination compared to infection naïve participants [80].

## Data Availability

All data quoted is available within the public domain.
